# A user-friendly online tool for paleocoordinate calculation and 3D visualization

**DOI:** 10.1038/s41598-026-46309-z

**Published:** 2026-06-15

**Authors:** Noa Scholz-Murcia, Alejandro Rodríguez-Mena, Víctor Madarnás-Gómez, Antonio Monleón-Getino

**Affiliations:** 1 BIOST3, Research Group in Biostatistics, Data Science and Bioinformatics, Department of Genetics, Microbiology and Statistics, Faculty of Biology, University of Barcelona (UB), Barcelona, Spain; 2 Institut de Recerca de la Biodiversitat (IRBio), Universitat de Barcelona, Barcelona, Spain

**Keywords:** Mathematics and computing, Solid Earth sciences

## Abstract

To understand the distribution of continents throughout geological history, researchers rely on Global Plate Models (GPMs), which describe the movement of Earth’s tectonic plates over time. However, the complexity and variability of these models may limit their accessibility to non-specialist researchers. To address this challenge, we developed Paleocoordinates Calculator (PACA). PACA is a web-based graphical interface built on the *palaeoverse* R package, which in turn queries the GPlates Web Service to perform plate-tectonic rotations. PACA enables researchers to upload georeferenced data and select from a range of GPMs to obtain reconstructed paleocoordinates without requiring programming skills or specialized software. PACA also enables three-dimensional visualization of reconstructed data integrated with Scotese’s paleogeographic maps. Technical validation shows near-perfect agreement with GPlates desktop software, with median spatial errors under 17 m, a Concordance Correlation Coefficient (CCC) of 1.000, and no systematic biases (Wilcoxon tests, *p* > 0.05) across all models. By simplifying access to paleogeographic rotation, PACA streamlines workflows and promotes transparency and reproducibility in Earth science research.

**Supplementary Information:**

The online version contains supplementary material available at 10.1038/s41598-026-46309-z.

## Introduction

Understanding the distribution of continents throughout geological history represents one of the most complex challenges in Earth sciences. Paleogeography aims to reconstruct the geographical context of continents across the geological timescale^1^. The continuous effort to trace the complex spatial and temporal history of Earth has fostered the development of a wide array of techniques and methodologies^2–7^. These advancements not only deepen our understanding of the past but also enhance our comprehension of the present and even inform predictions of future planetary changes.

### Historical context

Over centuries, the ambition to map and understand our planet drove significant technological revolutions, leading to cartography’s current high level of accuracy. The early 20th century’s view of Earth as a geographically static entity was challenged by Alfred Wegener’s 1915 publication^8^. His revolutionary theory of continental drift was supported by diverse geographical, geological, tectonic, paleoclimatic, and paleontological evidence^9^. This idea was integrated into the unifying theory of Plate Tectonics in the 1960s, a synthesis that completely reshaped our understanding of Earth as an active, dynamic planet^10–12^. The acceptance of Plate Tectonics created an urgent need to accurately reconstruct the position of continents and oceans throughout Earth’s history.

### Global plate models: foundations of paleogeographic reconstruction

The accuracy of paleogeographic reconstructions relies fundamentally on Global Plate Models (GPMs)^13^. They constitute quantitative kinematic frameworks that describe the relative and absolute motions of Earth’s lithospheric plates over geological time^13^. These models are built from complex datasets and algorithms derived through the integration of multiple geophysical sources. Key inputs include the patterns and spreading rates of oceanic crustal magnetic anomalies^14^, the geometry of oceanic transform faults^12^, paleomagnetic signatures preserved in ancient rocks^15^, and the trajectories of mantle hotspots^16^. By applying Euler’s rotation theorem, GPMs encode the motion of the tectonic plates across Earth’s spherical surface^17–19^.

Paleomagnetism provides a key constraint for reconstructing continental paleolatitude and relative orientation through time^20,21^. If sufficient data are available from a single continent, its motion through time can be traced via an Apparent Polar Wander Path (APWP)^22^. When integrated on a global scale, multiple continental APWPs yield a Global Apparent Polar Wander Path (GAPWaP), offering a more robust framework for describing continental evolution^13,19^.

However, paleomagnetism cannot directly determine absolute paleolongitude^22^. To overcome this limitation, several methods have been developed^13^. Among them, the Plume Generation Zone (PGZ) method infers absolute paleolongitude by linking the surface expression of Large Igneous Provinces and kimberlites to apparently stable deep mantle structures^23^. The orthoversion model posits that successive supercontinents assemble orthogonally over mantle downwellings, offering a kinematic constraint on their longitudinal placement^24^. Despite inherent limitations—such as assumptions about deep mantle stability, the difficulty of separating continental motion from True Polar Wander (i.e., “the rotation of the Earth’s lithosphere and mantle with respect to the spin axis”)^19^, and dependence on robust geological datasets^13^—these methods provide essential frameworks for inferring paleolongitude, and are expected to improve with further research.

A diversity of recognized GPMs is employed within the scientific community^5,7,13,19,25–27^. These models stem from varying interpretations of geophysical data, distinct construction methodologies, and underlying assumptions about absolute reference frames. Consequently, the technical complexity and inherent variability of these models introduce uncertainty into paleogeographic analyses while also posing a considerable accessibility barrier for non-specialist researchers.

### An accessible solution for modern paleogeographic research

For many researchers in fields such as paleobiology or paleoecology, integrating paleocoordinates into their analyses poses a significant challenge. Determining a past location requires access to complex models and the ability to apply appropriate geophysical rotations, often demanding efficient computational tools to process large volumes of data. Additionally, existing databases containing paleocoordinate data (e.g., The Paleobiology Database^28)^ do not often provide specification of which GPM was utilized. Consequently, this technological barrier prevents researchers from fully leveraging paleocoordinates’ potential in their studies.

To address these challenges and democratize access to high-quality paleogeographic reconstructions, we developed **Paleocoordinates Calculator (PACA)**^29^, a web interface.

Rather than introducing a new plate-tectonic model, PACA lowers the technical barrier to accessing established Global Plate Models by providing a **web-based graphical interface** to existing, community-validated reconstruction models. PACA enables users, without requiring programming skills or downloading any additional software, to upload occurrence data in comma-separated values (CSV) format, and obtain reconstructed paleocoordinates for leading and widely recognized GPMs. PACA also offers the functionality to **visualize and download** results directly onto **three-dimensional paleomaps** from Scotese’s PALEOMAP Project^5^, which are a visual standard in the paleoscientific community.

A comparative overview of PACA’s capabilities alongside existing solutions for paleocoordinate calculation is provided in Supplementary Table 1, which has been deposited in the **Zenodo data repository**^30^.

With its intuitive interface and focus on accessibility, PACA provides a practical tool for the paleoscientific community, facilitating the use of established reconstruction models. By streamlining workflows for non-specialists, it contributes to the consistent application of paleogeographic analyses across the Earth sciences.

## Methods

### Tool architecture and core technologies

Paleocoordinates Calculator (PACA) is a web-based graphical interface designed for paleocoordinate reconstruction. The tool’s computational backend relies on the *palaeoverse*^31^ R package, specifically its palaeorotate() function^31^. This core function utilizes five GPMs to reconstruct paleocoordinates: PALEOMAP^5^, GOLONKA^7^, MERDITH2021^13^, TorsvikCocks2017^19^ and MATTHEWS2016_pmag_ref^25^. Accordingly, PACA does not introduce a new plate-tectonic model, but relies on the *palaeoverse* R package, which accesses the GPlates Web Service^32^ to apply Euler rotations from these five published GPMs.

The web interface is built with Vite and React as a routed application, communicating via REST with the computational backend to request paleocoordinate rotations and displaying results in an interactive 3D viewer alongside searchable, exportable tables, which facilitates a dynamic and user-friendly experience.

To execute a reconstruction, the palaeorotate() function requires **eight arguments**: a **dataframe** of fossil occurences, the **spatial coordinates (lng and lat)**, the **geological age**, the **tectonic model**, the **reconstruction method** and the **parameters for uncertainty and rounding**.

The function only accepts a **single age** for each record. Following the function’s design, we used the arithmetic mean of the minimum and maximum ages (in millions of years, Ma) as the input age. PACA automatically calculates the arithmetic mean age of each occurrence’s age range (from the age_min and age_max columns) using R’s rowMeans() function^33^, and supplies this value as the ‘age’ argument in the palaeorotate() function.

The function offers two distinct **methodologies** for paleocoordinate reconstruction: **The ‘grid’ method** provides efficient estimations linking modern coordinates and age estimates to a set of pre-calculated paleocoordinates, generated from an equal-area hexagonal grid (with ~ 100 km spacings). In contrast, **the ‘point’ method** reconstructs each coordinate individually by querying the GPlates Web Service. The latter performs a continuous mathematical rotation on the input coordinates, meaning its precision is not limited by a grid cell size but is instead determined by the accuracy of the plate boundary polygons within the GPM itself. This method offers higher precision by avoiding the spatial aggregation inherent to the ‘grid’ approach. To ensure that users are provided with the highest possible spatial accuracy, **PACA is built exclusively upon the ‘point’ method**.

The quantification of inter-model variability is implemented in PACA by setting the **‘uncertainty’ argument** of the palaeorotate() function to **TRUE**. Detailed information regarding this quantification is provided in Sect. Methods, specifically in the subsection Model-Variability Quantification.

The **‘round’ parameter** is set to 3 by default, which optimizes computational efficiency by reducing the number of unique coordinate pairs while maintaining sufficient spatial precision for deep-time reconstructions.

Summarizing, PACA accepts present-day WGS84 coordinates as input and processes them via the palaeorotate() function, which queries the Gplates Web Service using five GPMs to reconstruct paleocoordinates, returning the results in tabular format as geographic latitude–longitude values (in decimal degrees).

High-resolution three-dimensional models representing different geological epochs were constructed by integrating paleogeographic map data obtained in JPG format from the reconstructions of Scotese^5^. The digital maps were georeferenced, converted to GeoTIFF, and subsequently projected as textures onto UV spheres using Blender. This methodology enabled the accurate placement of paleocoordinate points on each 3D model. Each paleomap was assigned to a specific geological period following the 2024 International Chronostratigraphic Chart^34^. The resulting globes provide a robust spatial framework for mapping paleogeographic data and visualizing location changes throughout deep time. The scripts for georeferencing, compressing, and converting raster map data into 3D visualizations have been deposited in the **Zenodo repository**^30^.

### Data input and workflow

**Fig. 1:**
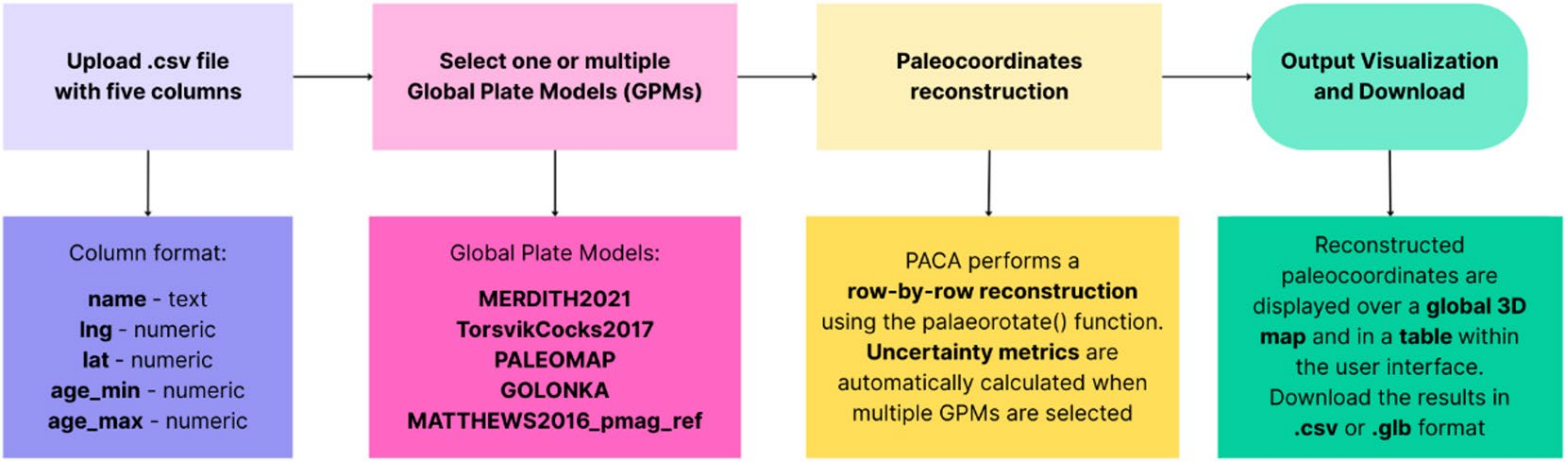
PACA workflow for paleocoordinate reconstruction.

The workflow in PACA is designed to be simple and intuitive (Fig. 1). The user uploads occurrence data as a CSV file, which must include five specific columns:

- **name** – Unique identifier for each record, in text format (e.g., *Sample_1*).
- **lng** – Longitude, expressed in decimal degrees according to WGS84 (e.g., *− 3.688344*).
- **lat** – Latitude, expressed in decimal degrees according to WGS84 (e.g., *40.453054*).
- **age_min** – Minimum age in millions of years (Ma) (e.g., *137.05*).
- **age_max** – Maximum age in millions of years (Ma) (e.g., *143.1*).

Within the interface, users can select **multiple GPMs for simultaneous reconstruction (up to all five available models).** If the input CSV contains blank values, entries that cannot be recognized as numeric (e.g. NA), commas instead of decimal points, or implausible values (e.g., latitudes outside the range − 90° to + 90°), PACA generates an automatic message indicating that an error occurred during the reconstruction process (e.g. *‘Invalid CSV format. Your file must contain these columns: lat*,* lng*,* age_min*,* age_max’*). Upon executing the tool, PACA processes each row of the input file and displays the reconstructed paleocoordinates within the application interface. Reconstructed paleocoordinates can be downloaded as a CSV file and a GLB file for visualization in 3D software.

For a detailed description of how to use PACA, please refer to the User Guide provided in the Zenodo Repository^30^.

### Global plate models

The five GPMs implemented in palaeorotate() —and therefore in PACA—are summarized in Table 1, highlighting their temporal coverage, reference frames, and reconstruction scope.

The reconstruction process is underpinned by Euler’s fixed-point theorem. This theorem states that the displacement of any rigid body on a sphere—such as a tectonic plate—can be described as a single rotation around a specific axis^17,18^. This axis is defined by a rotation (Euler) pole, which specifies a latitude, a longitude, and a rotation angle^19^. Building on this principle, past positions of input coordinates are reconstructed by applying the rotations defined by Euler poles from the GPMs.

**Table 1:** Characteristics of the GPMs implemented in PACA (via the palaeorotate() function).

Model identifier		Timespan (Ma)	Reference frame	Reconstructed lithosphere
PALEOMAP^5^		1100–0	Based on paleomagnetism + geological evidence, no mantle reference	Full-plate model (reconstructs both continental and oceanic lithosphere)
GOLONKA^7^		540–0	Paleomagnetic GAD reference frame updated from *Torsvik & van der Voo (2002)*^36^	Full-plate reconstruction (continents + oceans, cratons + terranes, oceanic plates via relative plate rotations)
MERDITH2021^13^		1000–0	Paleomagnetic (no mantle frame, no TPW correction)	Full-plate model (reconstructs both continental and oceanic lithosphere)
TorsvikCocks2017^19^		540–0	Paleomagnetic hybrid (PM-HYBRID / GMHRF), TPW-corrected, includes multiple hotspot frames (Pacific, Atlantic, Indian)	Full-plate reconstruction (continents + oceans, cratons + terranes, oceanic plates via hotspot rotations)
MATTHEWS2016_pmag_ref^25^		410–0	Multiple (e.g., TPW-corrected paleomagnetic, global moving hotspot, fixed Pacific hotspot)	Full-plate model (reconstructs both continental and oceanic lithosphere)

GPMs: Global Plate Models. TPW: True Polar Wander. PM: Paleomagnetic. GMHRF: Global Moving Hotspot Reference Frame. GAD: geocentric axial dipole.

The GPMs listed in Table 1 provide rotation parameters for each tectonic plate. They are distributed as **GPlates rotation files (.rot**,** .gpml)**, enabling continuous reconstructions. This involves a set of finite rotations defined at a given **temporal resolution** (often 1 Ma). Between these discrete steps, GPlates interpolates plate motion, allowing reconstruction at any intermediate time.

Each GPM is defined within a specific **reference frame**, a fixed coordinate system used to describe the absolute or relative motion of tectonic plates over time. Reference frames may be based on paleomagnetic data (either simple or corrected for True Polar Wander), fixed or moving hotspot frameworks, hybrid approaches combining paleomagnetic and hotspot data, or paleomagnetic reconstructions constrained by geological evidence.

The GPMs employ different approaches to reconstruct continental and oceanic lithosphere. Reconstructions for the Late Mesozoic and Cenozoic (0–200 Ma) are well constrained by direct geophysical data, including marine magnetic anomalies, seafloor ages, and fossil ridge traces. For older intervals (> 200 Ma, Late Triassic and earlier), oceanic crust has largely been subducted and is no longer preserved. Consequently, reconstructions of oceanic lithosphere for these periods are indirect or synthetic, relying on paleomagnetic data, continental fits, and inferred patterns of ocean expansion.

### Model-variability quantification

In addition to generating paleocoordinates, a key feature of PACA is the quantification of inter-model variability. When the user selects two or more GPMs for a reconstruction, PACA automatically returns two metrics for each input, both calculated by the palaeorotate() function^31^:

- **Paleolatitudinal range**: The difference in degrees of latitude between the maximum and minimum paleolatitude values obtained from the selected models.
- **Maximum geographic distance (km)**: The maximum great-circle (orthodromic) distance between any pair of reconstructed coordinates for a single input, computed internally using the distGeo() function from the *geosphere* R package^37,38^.

### Visualization and data export

Upon processing, PACA generates a tabular dataset containing reconstructed paleocoordinates, and associated model-variability metrics. Unlike the default output of the palaeorotate() function—which generates two distinct columns (paleolongitude and paleolatitude) for every selected model within a single row per entry—PACA provides a vertically integrated table. In this structure, each input record is represented in a separate row for each selected GPM. This results in a streamlined table with only two universal paleocoordinate columns (p_lng, p_lat) and a model column to differentiate the reconstructions. This format optimizes the dataset for direct use in statistical analysis and visualization. The resulting table includes the following output columns:

- **age_mean** – The arithmetic mean age between the minimum and maximum ages provided by the user (e.g., *140.075*).
- **p_lng** – Reconstructed longitude in geographic degrees (e.g., *23.834*).
- **p_lat** – Reconstructed latitude in geographic degrees (e.g., *26.597*).
- **period** – The geological period or boundary to which each record belongs (e.g., *Jurassic–Cretaceous Boundary*).
- **model** – The model used to calculate each record (e.g., *MERDITH2021*).
- **range_p_lat** – Paleolatitudinal range (e.g., *10.425*).
- **max_dist** – Maximum geographic distance in km (e.g., *2220.021*).

The generated results can be **visualized** directly within the PACA interface, either in tabular form or on an interactive 3D globe. On the globe, the reconstructed paleocoordinates can be overlaid onto a selection of high-resolution 3D paleogeographic maps representing different geological stages from Scotese’s PALEOMAP Project^5^, with one map provided for each geological period corresponding to the input records, as defined according to the International Chronostratigraphic Chart^34^.

For **data export**, PACA provides two options: tabular results—including paleocoordinates and their associated metrics—in **CSV format**, and a **GLB file** for visualization in 3D software. These formats facilitate inclusion in publications or offline analysis.

### Technical validation

The technical validation of PACA was conducted **to ensure consistency between the PACA web interface and traditional GPlates desktop software**^39^**workflow**. Since PACA queries the GPlates Web Service via the palaeorotate() function (*palaeoverse* R package), this procedure serves as a consistency check between **automated API-based rotations (PACA)** and the **traditional desktop-based reconstruction workflow (GPlates)**. Specifically, PACA’s outputs were compared against paleocoordinates calculated individually in GPlates desktop using the original rotation (.rot) and plate polygon (.gpml) files from each tectonic model’s primary publication.

External cross-validation was performed using a test dataset of 142 entries (derived from 30 fossil sites) with present-day coordinates, globally distributed and spanning a wide range of geological ages. To address the spherical and circular nature of paleocoordinates, consistency was assessed using three robust metrics:

1. **Great Circle Distance (Haversine)**: Used to calculate the physical spatial error in kilometers, accounting for Earth’s curvature to ensure that displacement is measured as true physical distance.
2. **Lin’s Concordance Correlation Coefficient (CCC)**: Used to evaluate the agreement between interfaces by measuring how closely the data follows the 45-degree line of identity, assessing both precision and accuracy.
3. **Wilcoxon Signed-Rank Test**: A non-parametric test applied to angular differences in paleolongitude (corrected for the 180° antimeridian wraparound) to identify potential systematic biases.

All statistical analyses were conducted in R version 4.3.2.^33^.

Occasionally, the palaeorotate() function—and, consequently, PACA—was unable to calculate certain paleocoordinates, resulting in 7 excluded cases (4.7% of the 150 potential reconstructions). Independent verification confirmed that these points could not be reconstructed in the GPlates desktop environment either, as they stem from inherent model limitations. These exclusions are:

- **Temporal Scope Limits (2 cases)**: *A. canadensis* (508 Ma) and *E. kingii* (503 Ma) could not be reconstructed using MATTHEWS2016_pmag_ref, as the specimen ages exceed the model’s 410 Ma maximum limit.
- **Plate Ephemerality (5 cases)**: The specific Plates assigned to the fossils do not exist for the requested reconstruction ages: *A. canadensis* (508 Ma) could not be reconstructed in MERDITH2021 because Plate ID 154 is only defined back to 410 Ma. *E. kingii* (503 Ma) could not be reconstructed in TorsvikCocks2017 because Plate ID 153 is only defined back to 300 Ma. *O. megalodon* (10.1 Ma) could not be reconstructed in GOLONKA (Plate ID 230 ends at 5.4 Ma), MERDITH2021 (Plate ID 229 ends at 5 Ma), and MATTHEWS2016_pmag_ref (Plate ID 224 ends at 3 Ma).

In all 7 cases, the GPlates Web Service returned identical null values (999.99). Consequently, the statistical analyses were initially performed using the 143 reconstructions for which a direct comparison between automated and manual workflows was viable. Additionally, one record (*I. communis* at 194 Ma for the TorsvikCocks2017 model) was excluded during the initial validation. In the manual GPlates desktop workflow, a Plate ID was assigned to this specimen despite it falling slightly outside the defined plate boundary. This assignment led to a geometric distortion of ~ 2,000 km upon reconstruction. To ensure a rigorous comparison of the software’s intrinsic computational accuracy without the interference of manual assignment errors, this outlier was removed. Consequently, the **final validation was conducted on 142 successful reconstructions**.

## Results

The cross-validation yielded a near-perfect alignment between the PACA web interface and GPlates desktop workflow across all tectonic models (Table 2).

The median spatial error ranged from 0.00625 km to 0.0168 km, representing a physical deviation of approximately 6.25 to 16.8 m.

The Concordance Correlation Coefficient (CCC) reached 1.000 for all five tectonic models tested (GOLONKA, MATTHEWS2016_pmag_ref, MERDITH2021, PALEOMAP, and TorsvikCocks2017).

Furthermore, the Wilcoxon signed-rank test resulted in *p*-values ranging from 0.173 to 0.540, well above the 0.05 significance threshold.

**Table 2:** Technical validation metrics comparing PACA (via GPlates Web Service) and GPlates Desktop workflow across five tectonic models (*N* = 142).

Tectonic model	N	Median spatial error (km)	CCC (Longitude)	Wilcoxon test (p-value)
GOLONKA	29	0.0117	1.000	0.419
MATTHEWS2016_pmag_ref	27	0.00625	1.000	0.446
MERDITH2021	28	0.0102	1.000	0.182
PALEOMAP	30	0.0146	1.000	0.173
TorsvikCocks2017	28	0.0168	1.000	0.540

## Discussion

### Reconstruction age

PACA assigns the arithmetic mean of the user-specified minimum and maximum ages for rotation to each occurrence. This provides a single representative age per record, as required by the palaeorotate() function. The arithmetic mean of the age range was chosen as a representative value, as it equally balances the minimum and maximum ages without bias toward either extreme. As this approach does not capture the full temporal range, resulting paleocoordinates represent the central position of the age interval. We acknowledge that this approach simplifies temporal uncertainty, particularly for intervals of rapid plate motion, and may slightly affect the precision of reconstructed palaeocoordinates. Users interested in exploring the full range of temporal variability can perform additional reconstructions at the extremes of the age interval by assigning the same value to both the minimum and maximum age columns for each occurrence (i.e., setting the earliest value to both age_min and age_max columns to reconstruct the earliest position, or the latest value to both columns for the latest position). This generates separate paleocoordinates for each extreme of the age range. Similarly, users can explore any other representative value within the age range by calculating it independently and entering the same value in both columns prior to reconstruction.

### Applications for model-variability quantification

In addition to generating paleocoordinates, PACA provides two metrics to assess inter-model variability: paleolatitudinal range, and maximum geographic distance in kilometers. Both metrics are calculated by the palaeorotate() function, and may be useful for interpreting paleogeographic results, as they assess the range of variation across alternative GPMs. For example, researchers could determine whether a locality consistently remained within a specific climatic zone (e.g., tropics) or biogeographic province; a large range in either metric may indicate a region with a complex tectonic history, warranting caution in interpretation; these values may also be correlated with paleoclimate data to assess whether a species was a climatic generalist or specialist, or to determine whether an organism’s range was confined to a single plate; finally, these values could be related to macroevolutionary patterns, such as speciation or extinction events.

### Technical validation

The results of the technical validation confirm that PACA is a **high-fidelity proxy** for traditional GPlates desktop workflows. The observed **median spatial errors**—ranging from 6.25 m (0.00625 km) in the MATTHEWS2016_pmag_ref model to 16.8 m (0.0168 km) in TorsvikCocks2017—fall several orders of magnitude below the inherent uncertainties of global tectonic models, which typically operate on scales of tens to hundreds of kilometers. This minimal discrepancy likely stems from slight versions mismatches or differences in coordinate precision within the rotation (.rot) and polygon (.gpml) files between the local GPlates desktop setup and the GPlates Web Service API. The perfect **Concordance Correlation Coefficient** (CCC = 1.000) across all five tectonic models and the **non-significant p-values from the Wilcoxon tests** (*p* > 0.05) demonstrate that PACA does not introduce systematic biases or “drift” during the automated query process. Furthermore, rather than relying solely on failing to reject the null hypothesis, true practical equivalence is established by the magnitude of the effect size: the median spatial error (< 17 m) is geometrically negligible for global tectonic models.

This high level of agreement, maintained across 142 successful reconstructions spanning from the Cenozoic to the Paleozoic, confirms that the tool is mathematically consistent with the traditional desktop-based reconstruction workflow.

The **exclusion of 8 cases** stemming from inherent geological constraints ensures that the validation metrics reflect only valid comparison data. These instances represent model-specific “data gaps” rather than algorithmic errors, allowing for a reliable assessment of PACA’s consistency in automating traditional workflows without introducing computational bias.

A noteworthy finding during the validation process was the sensitivity of results to the **specific version of the rotation files** used. Matthews et al. (2016)^25^ derive paleolatitudes directly from paleomagnetic data, whereas paleolongitudes are not constrained by geological records and are determined by relative plate motions and the absolute reference frame applied. The GPM has been released in multiple reference frames, including: an absolute reference frame linked to the **mantle** (TPW-corrected, 0–230 Ma); a **hybrid** reference frame combining **moving hotspots** for 0–70 Ma with a TPW-corrected **paleomagnetic** reference frame for 70–230 Ma; and a purely **paleomagnetic** (TPW-corrected) reference frame for 230–410 Ma. It is plausible that the reference frame used in PACA—accessed via the palaeorotate() function, which queries the GPlates Web Service—differ slightly from the initial reference frame directly applied within the GPlates desktop software. In such frameworks, small differences in how the Euler rotations are applied result in substantial longitudinal shifts, particularly in polar regions where meridians converge. Preliminary tests using the most recent supplementary files from the Matthews et al. (2016) model (updated repositories) showed significant deviations from the PACA outputs generated by the GPlates Web Service API via palaeorotate(). However, full consistency was achieved when using the model’s earlier “pmag_ref” version. This indicates that the **GPlates Web Service maintains an unupdated paleomagnetic reference frame for this specific model**, highlighting that researchers must ensure the synchronization of rotation (.rot) and polygon (.gpml) files between GPlates desktop software and PACA to maintain reproducibility.

By bridging the gap between automated R-based workflows and established desktop standards, PACA provides a validated, reproducible, and computationally efficient method for large-scale paleogeographic analyses. This integration allows researchers to process high volumes of data without compromising the geometric rigor required in tectonic research, ensuring that automated reconstructions remain as reliable as traditional manual workflows.

### Reproducibility and long-term stability

PACA relies on the dynamic GPlates Web Service for its calculations—queried via the palaeorotate() function—, and temporary service downtime or future updates could potentially affect its **availability** or introduce **version-related differences**. Since PACA retrieves data from the GPlates Web Service, the results are **dependent on the current version of the rotation files (.rot and .gpml)**. Consequently, a reconstruction performed today might yield slightly different coordinates than one performed in the future if the GPlates API updates its internal rotation files to reflect more recent plate tectonic refinements. 

To ensure validation and long-term consistency, we provide a static benchmark in the Zenodo repository^30^, which includes the **test dataset** used for validation and the corresponding **paleocoordinate outputs** generated by this specific version of PACA (v1.0.1) and reconstructed with GPlates desktop software. Also, the output CSV file downloaded by the user includes **metadata** detailing the **specific GPMs used** for the reconstruction (column ‘model’), as well as **the date and time of the rotation** (in the file name). In addition, the **source code of PACA** is **openly available**, together with the **scripts and files for 3D visualization**. These files serve as a permanent reference, allowing the functionality of the tool to be verified in the future, even if the underlying web service evolves or the GPMs versions change. Alternative approaches—such as incorporating the ‘grid’ method of the palaeorotate() function—could be implemented if necessary to ensure the web interface continues to function correctly and provide reliable results.

Users can accurately report the **methodology** employed in their studies. To manage the GPM version-related uncertainty, we provide the following guidance for users:

- **Reporting**: Authors should explicitly state the access date of the PACA interface in their methodology, as this serves as a proxy for the GPlates API version at that time.
- **Archiving**: Users should treat the downloaded CSV as the ‘raw data’ of their paleogeographic analysis. Since the GPlates Web Service does not currently allow users to select specific older versions of a tectonic model, the PACA output file is the only static record of the specific rotation applied. Users should document the specific GPM selected and the reconstruction age.

## Supplementary Information

Below is the link to the electronic supplementary material.

Supplementary Material 1

## Data Availability

The PACA web interface (v1.0.1) is accessible at https://biost3.bio.ub.edu/paca. The source code for the Paleocoordinates Calculator (PACA v1.0.1) is openly available in the Zenodo repository^30^ at https://zenodo.org/records/19332776 under the GNU General Public License v3 (GPLv3). This repository includes: validation_data_original.xlsx: Complete test dataset used for study validation, including 30 fossil sites with present-day coordinates, globally distributed and spanning a wide range of geological ages. validation_data_paleocoordinates.xlsx: Dataset of 150 paleocoordinates, including NAs, calculated manually using GPlates and automatically with PACA. validation_references.pdf: Detailed bibliography of the test dataset, referencing original sources. SUPPLEMENTARY TABLE 1.pdf: Detailed comparison between PACA and other apps. user_guide.pdf: Comprehensive step-by-step user guide for using Paleocoordinates Calculator. template_sample_case.csv: Downloadable template to help users align their data with the required structure. results_sample_case_PACA.csv: A sample output file that allows users to see exactly how the reconstructed results will be formatted. .glb paleomaps: 3D paleogeographic maps adapted from the Scotese PALEOMAP Project^5^. The collection of open-source Python and Blender scripts used for georeferencing, raster processing, and 3D map conversion is archived in a separate Zenodo repository^35^ at https://doi.org/10.5281/zenodo.17642407, under the MIT License. The 3D .glb files provided in our repository^30^ constitute an adaptation (2D to 3D conversion, as described in the Methods section) of the original maps from the PALEOMAP project^5^ and are licensed under CC BY 4.0. To view a copy of this license, visit https://creativecommons.org/licenses/by/4.0/.
