# Supplementary material for: A user-friendly online tool for paleocoordinate calculation and 3D visualization
Source: Sci Rep. 2026 Jun 15;16:18518. doi: 10.1038/s41598-026-46309-z (PMC13269751; doi:10.1038/s41598-026-46309-z)
Supplement: Supplementary file 1 — Supplementary Material 1 [file 41598_2026_46309_MOESM1_ESM.pdf]

A user-friendly online tool for paleocoordinate calculation and 3D visualization

Noa Scholz-Murcia[1,2], Alejandro Rodríguez-Mena[1,2], Víctor Madarnás-Gómez[1,2], Antonio Monleón-Getino[1,2]

[1] BIOST3, Research Group in Biostatistics, Data Science and Bioinformatics, Universitat de Barcelona (UB), Barcelona, Spain.
[2] Department of Genetics, Microbiology and Statistics, Faculty of Biology, Universitat de Barcelona (UB), Barcelona, Spain.
Corresponding author: E-mail: scholz@ub.edu (N. S.)

SUPPLEMENTARY TABLE 1

Supplementary Table 1. Comparison of representative existing paleocoordinate reconstruction tools and PACA.

Tool	GPlates Desktop Software[1]	'Palaeorotate' ('Palaeoverse' R package)[2]	PAMS paleolocation.org[3]	paleolatitude.org[4]	ODSN[5]	PACA (This Study)
**Access**	Desktop Software	R[6] or Rstudio[7]	Web Platform	Web Platform	Web Platform	Web Platform
**Programming knowledge required**	Not strictly required (optional via Python[8])	Yes	No	No	Yes	No
**Learning curve**	High	Low	Low	Low	High	Low
**Batch data upload**	Yes	Yes	No	Yes	Yes	Yes
**Data input format**	Specialized (.rot, .gpml, .gdal, .csv, .shp)	Tabular dataframe	Simple (single entry)	Excel/CSV	Command-line strings	CSV
**Timespan (Ma)**	2500–0 Ma (Depending on the GPM, practical use often limited to ~1000 Ma)	1100–0 Ma (Depending on the GPM)	~520–0 Ma	550–0 Ma	150–0 Ma	1100–0 Ma (Depending on the GPM)
**GPMs utilized**	Any in .rot format (or convertible to it)	PALEOMAP[9] GOLONKA[10] MERDITH2021[11] TorsvikCocks2017[12] MATTHEWS2016_pmag_ref[13]	PLATES Project[14]	Limited to paleomagnetic based GPMs[(15-18)]*	Wilson (1989) terranes[19]; rotations by Soeding[20] (maps compiled in Hay et al. (1999)[21])	PALEOMAP[9] GOLONKA[10] MERDITH2021[11] TorsvikCocks2017[12] MATTHEWS2016_pmag_ref[13]
**Paleomap visualization and download**	Yes	Other packages required	Yes	No	Yes	Yes
**Uncertainty Calculation**	Yes (via pyGPlates/ GPlately and post-processing in Python[8])	Yes (directly)	No	No	No	Yes (directly)

PAMS: Paleolocation Mapping Service. ODSN: Ocean Drilling Stratigraphic Network. PACA: Paleocoordinates Calculator. CSV: Comma-separated values format. GPM: Global Plate Model. * Models restricted to a specific set of APWP.

References for Supplementary Table 1

1. Müller, R. D. *et al.* GPlates: building a virtual Earth through deep time. *Geochem. Geophys. Geosyst.* **19**, 2243–2261 (2018).

2. Jones, L. A. et al. palaeoverse: a community-driven R package to support palaeobiological analysis. *Methods Ecol. Evol.* **14**, 2205–2215 (2023).

3. Urban, T. & Hardisty, F. Developing PAMS—a paleolocation web service (#1305). In *26th International Cartographic Conference Proceedings* 308 (2013).

4. van Hinsbergen, D. J. J. *et al.* A paleolatitude calculator for paleoclimate studies. *PLoS ONE* **10**, e0126946 (2015).

5. Ocean Drilling Stratigraphic Network (ODSN). ODSN Plate Tectonic Reconstruction Service. https://www.odsn.de/odsn/services/paleomap/paleomap.html (2011).

6. R Core Team. *R: A Language and Environment for Statistical Computing*. R Foundation for Statistical Computing https://www.R-project.org/ (2023).

7. Posit team. *RStudio: Integrated Development Environment for R.* (Posit Software, 2025).

8. Python Software Foundation. *Python Language Reference*, Version 3.13. (2024).

9. Scotese, C. R. Tutorial: PALEOMAP paleoAtlas for GPlates and the paleoData plotter program. *PALEOMAP Project, Technical Report* (2016).

10. Wright, N., Zahirovic, S., Müller, R. D. & Seton, M. Towards community-driven paleogeographic reconstructions: integrating open-access paleogeographic and paleobiology data with plate tectonics. *Biogeosciences* **10**, 1529–1541 (2013).

11. Merdith, A. S. *et al.* Extending full-plate tectonic models into deep time: linking the Neoproterozoic and the Phanerozoic. *Earth-Sci. Rev*. **214**, 103477 (2021).

12. Torsvik, T. H. & Cocks, L. R. M. *Earth History and Palaeogeography*. (Cambridge Univ. Press, 2017).

13. Matthews, K. J. *et al.* Global plate boundary evolution and kinematics since the late Paleozoic. *Glob. Planet. Change* **146**, 226–250 (2016).

14. University of Texas Institute for Geophysics (UTIG). PLATES Project. https://www.ig.utexas.edu/research/projects/plates/

15. Besse, J. & Courtillot, V. Apparent and true polar wander and the geometry of the geomagnetic field over the last 200 Myr. *J. Geophys. Res*. **107**, 2300 (2002).

16. Kent, D. V. & Irving, E. Influence of inclination error in sedimentary rocks on the Triassic and Jurassic apparent pole wander path for North America and implications for Cordilleran tectonics. *J. Geophys. Res*. **115**, B10103 (2010).

17. Doubrovine, P. V., Steinberger, B. & Torsvik, T. H. Absolute plate motions in a reference frame defined by moving hot spots in the Pacific, Atlantic, and Indian oceans. *J. Geophys. Res.* **117**, B09101 (2012).

18. Vaes, B. *et al.* A global apparent polar wander path for the last 320 Ma calculated from site-level paleomagnetic data. *Earth-Sci. Rev.* **245**, 104547 (2023).

19. Wilson, K. M. *Mesozoic Suspect Terranes and Global Tectonics*. PhD thesis, Univ. of Colorado (1989).

20. Cox, A. & Hart, R. B. *Plate Tectonics: How It Works*. (Blackwell Scientific Publications, 1986).

21. Hay, W. W. *et al.* Alternative global Cretaceous paleogeography. In *The Evolution of Cretaceous Ocean/Climate Systems* (eds Barrera, E. & Johnson, C.) 1–47 (Geological Society of America, 1999).
